# High *Trypanosoma* spp. diversity is maintained by bats and triatomines in Espírito Santo state, Brazil

**DOI:** 10.1371/journal.pone.0188412

**Published:** 2017-11-27

**Authors:** Maria Augusta Dario, Cristiane Varella Lisboa, Luciana M. Costa, Ricardo Moratelli, Monique Pereira Nascimento, Leonora Pires Costa, Yuri Luiz Reis Leite, Martin S. Llewellyn, Samanta Cristina das Chagas Xavier, André Luiz Rodrigues Roque, Ana Maria Jansen

**Affiliations:** 1 Laboratório de Biologia de Tripanosomatídeos, Instituto Oswaldo Cruz, Fundação Oswaldo Cruz, Rio de Janeiro/RJ, Brazil; 2 Laboratório de Ecologia de Mamíferos, Universidade do Estado do Rio de Janeiro, Rio de Janeiro/RJ, Brazil; 3 Fiocruz Mata Atlântica, Fundação Oswaldo Cruz, Rio de Janeiro/RJ, Brazil; 4 Departamento de Ciências Biológicas, Centro de Ciências Humanas e Naturais, Universidade Federal do Espírito Santo, Vitória/ES, Brazil; 5 Institute of Biodiversity, Animal Health and Comparative Medicine, University of Glasgow, Glasgow, Scotland, United Kingdom; Universidad de Chile, CHILE

## Abstract

The aim of this study was to reevaluate the ecology of an area in the Atlantic Forest, southeast Brazil, where Chagas disease (CD) has been found to occur. In a previous study, immediately after the occurrence of a CD case, we did not observe any sylvatic small mammals or dogs with *Trypanosoma cruzi cruzi* infections, but *Triatoma vitticeps* presented high *T*. *c*. *cruzi* infection rates. In this study, we investigated bats together with non-volant mammals, dogs, and triatomines to explore other possible *T*. *c*. *cruzi* reservoirs/hosts in the area. Seventy-three non-volant mammals and 186 bats were captured at three sites within the Guarapari municipality, Espírito Santo state. Rio da Prata and Amarelos sites exhibited greater richness in terms of non-volant mammals and bats species, respectively. The marsupial *Metachirus nudicaudatus*, the rodent *Trinomys paratus*, and the bats *Artibeus lituratus* and *Carollia perspicillata* were the most frequently captured species. As determined by positive hemocultures, only two non-volant mammals were found to be infected by *Trypanosoma* species: *Monodelphis americana*, which was infected by *T*. *cascavelli*, *T*. *dionisii* and *Trypanosoma* sp., and *Callithrix geoffroyi*, which was infected by *T*. *minasense*. Bats presented *T*. *c*. *cruzi* TcI and TcIII/V, *T*. *c*. *marinkellei*, *T*. *dionisii*, *T*. *rangeli* B and D, and *Trypanosoma* sp. infections. Seven dogs were infected with *T*. *cruzi* based only on serological exams. The triatomines *T*. *vitticeps* and *Panstrongylus geniculatus* were found to be infected by trypanosomes via microscopy. According to molecular characterization, *T*. *vitticeps* specimens were infected with *T*. *c*. *cruzi* TcI, TcII, TcIII/V, and TcIV, *T*. *c*. *marinkellei* and *T*. *dionisii*. We observed high trypanosome diversity in a small and fragmented region of the Atlantic Forest. This diversity was primarily maintained by bats and *T*. *vitticeps*. Our findings show that the host specificity of the *Trypanosoma* genus should be thoroughly reviewed. In addition, our data show that CD cases can occur without an enzootic cycle near residential areas.

## Background

The *Trypanosoma* genus comprises flagellate species that can infect diverse animal species and are transmitted by hematophagous invertebrate hosts [1–2]. These parasites are divided into two biological groups based on their development in invertebrate hosts: Salivaria and Stercoraria [3–4]. *Trypanosoma* is composed of parasite species of medical and veterinarian importance, such as *Trypanosoma cruzi cruzi*, which is responsible for Chagas disease (CD) and *Trypanosoma brucei*, which is responsible for sleeping sickness in humans and nagana in cattle in Africa [5–6].

The *T*. *cruzi* clade includes *T*. *c*. *cruzi*, *T*. *c*. *marinkellei*, *T*. *dionisii* and *T*. *erneyi*; a group known as *T*. *rangeli*/*T*. *conorhini*, which consists of *T*. *rangeli*, *T*. *conorhini*, *T*. *vespertilionis* and trypanosome species isolated from terrestrial African mammals [7–9]. It also includes trypanosomes that have been isolated from Neotropical bats [10], Australian marsupials [11–13] and *T*. *livingstonei*, which was isolated from African bats [14]. All trypanosomes except for *T*. *c*. *cruzi* and *T*. *rangeli* are known to infect specific animal groups. There are two hypotheses for the origin of the *T*. *cruzi* clade: the first hypothesis, i.e., the southern supercontinent hypothesis [15], proposes that *T*. *c*. *cruzi* speciated in marsupials after the separation of South America from the Australian continent. The second hypothesis is known as the bat seeding hypothesis [16] and proposes that bats were the ancestral hosts of the *T*. *cruzi* clade. The latter hypothesis is gaining increasing support based on the description of trypanosome species in African mammals, American bats containing members of the *T*. *cruzi* clade and the low diversity of species of the *T*. *cruzi* clade in South American terrestrial mammals [7, 9, 10, 14].

*Trypanosoma cruzi cruzi* has a broad distribution in the New World, extending from the southern US to Chile and Argentina. As a heterogeneous parasite, seven discrete typing units (DTUs) are recognized: TcI to TcVI and TcBat [17–18]. In Brazil, after intradomiciliary transmission of the parasite, the primary route of infection is via oral transmission, and CD is re-emerging as a food-borne disease [19–20]. In Espírito Santo (ES) state in southeastern Brazil, residents are often in contact with triatomines, as these insects are attracted by light and frequently invade residences. The primary triatomine species in the region is *Triatoma vitticeps*, which exhibits high rates of *T*. *c*. *cruzi* infection [21]. In 2012, a child died from acute Chagas disease (aCD) acquired via oral transmission [22]. The child presented with a mixed infection of *T*. *c*. *cruzi* TcI, TcII, TcIII, and TcIV and *T*. *dionisii* [22]. During an initial investigation, we were not able to determine the reservoirs of *T*. *c*. *cruzi* in the area, since the dogs and small wild mammals in the surrounding area tested negative. None of the animals presented patent parasitemia or positive hemocultures, contrasting with the triatomines, which presented high *T*. *c*. *cruzi* infection rates. In addition, the domestic animals (dogs) were not infected, as shown by negative serological and parasitological tests [22]. These findings led us to hypothesize that the house-invading triatomines became infected with *T*. *c*. *cruzi* by feeding on wild hosts in an area distant from the peridomiciliar area where the human case of aCD occurred. To confirm this hypothesis, we decided to diagnose *T*. *c*. *cruzi* infection in wild mammals in two other areas that were farther away from the house where the case of aCD occurred to determine which mammal taxa maintain the enzootic transmission cycle of *T*. *c*. *cruzi*. Thus, our primary objective was to build upon the previous study to identify the mammal reservoirs of *T*. *c*. *cruzi* from which the *T*. *vitticeps* that invaded human dwellings were becoming infected with *Trypanosoma* spp.

## Materials and methods

### Ethics approval and consent to participate

The sampling procedures reported herein were authorized by the Brazilian Institute of the Environment and Renewable Natural Resources (IBAMA) under license no. 19037–1 for bats and license no. 10070–2 for non-volant mammals. The euthanasia and blood collection procedures met the guidelines set by the Federal Council of Veterinary Medicine, Resolution 1000 (11-05-2012), in accordance with Federal Law 11.794/2008. All procedures followed protocols approved by the Fiocruz Ethics Committee for Animal Research (L0015-07).

### Study area

**Fig 1:**
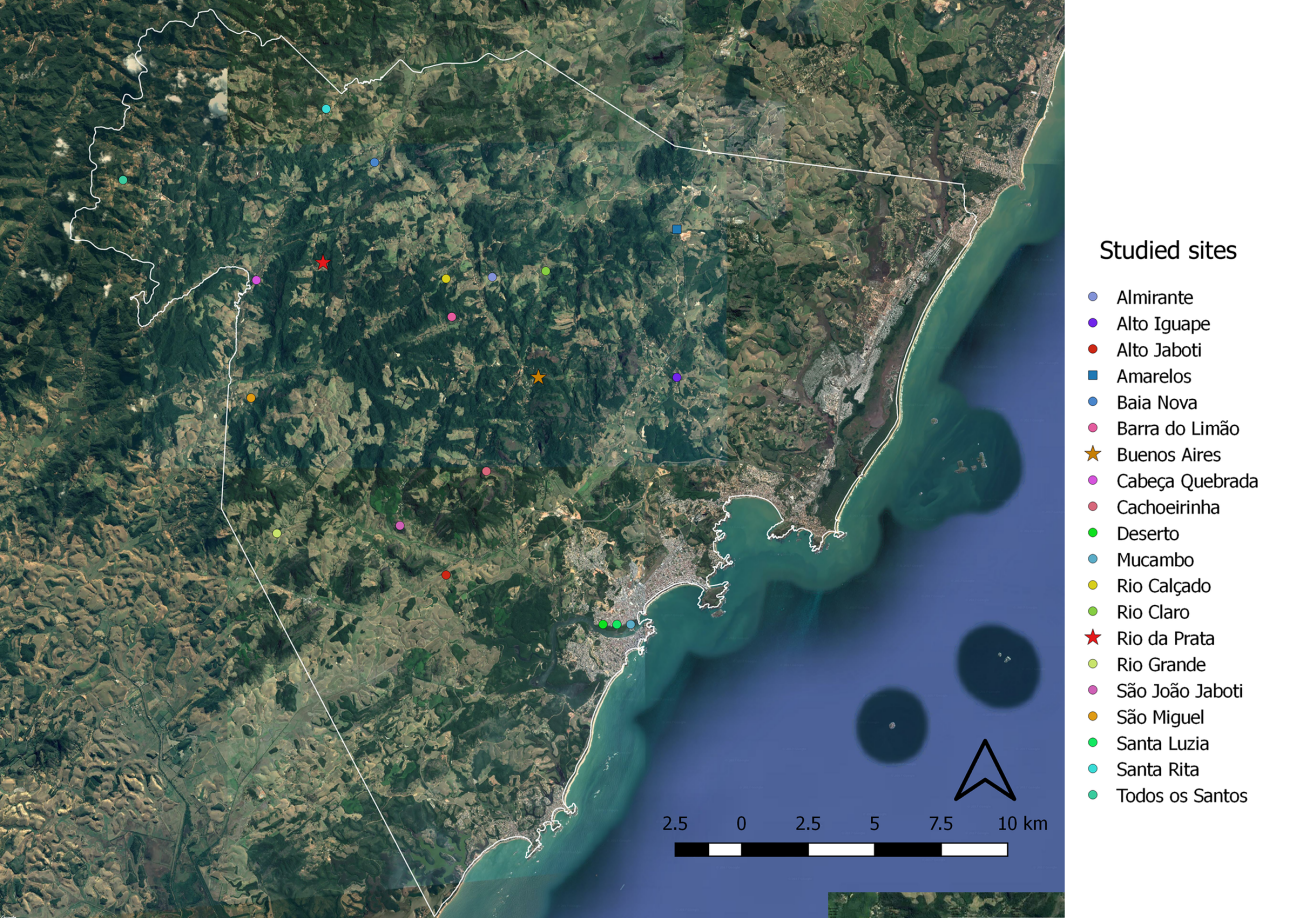
Guarapari municipality study locations. The stars represent locations where mammals and triatomines were captured, namely, Buenos Aires, an area where human dwelling invasion by adult triatomines (primarily by *T*. *vitticeps*) was reported by the residents; and Rio da Prata, the location where the aCD case occurred. The square represents Amarelos location, an area with no reports of human dwelling invasion by triatomines. The dots represent locations from which triatomines were received. Three of the triatomine collection locations (Deserto, Mucambo and Santa Luzia) were georeferenced by the municipality centroid, because we could not obtain the exact georeference of these collection site. (Source: Google Earth).

This study was conducted in three rural areas in the Guarapari municipality, located along the southeastern coast of Brazil, as described in [23] (Fig 1).

### Sylvatic small mammal capture

Four surveys were performed in the three studied areas, with two in the dry season (May 2014 and June 2015) and two during the rainy season (October 2014 and November 2015). For the small wild mammal captures, two linear transects consisting of 15 trapping stations that were 10 m apart were established at each study site. Each trapping station had one Sherman^®^ (H. B. Sherman Traps, Tallahassee, FL, USA) and one Tomahawk^®^ (Tomahawk Live Traps, Tomahawk, WI, USA) trap placed on the ground and in the understory tied to vines and lianas 1–1.5 m above the ground, when possible. The traps were baited with a mixture of pineapple and peanut butter and remained open for five consecutive nights during each sampling event, resulting in a trapping effort of 1,200 trap nights.

Bats were captured near the same transects using mist nets. Ten mist nets were placed in the surrounding forest and near food sources (fruit trees), shelters and flight routes, and they remained open for four hours after sunset. Bat captures were performed for two consecutive nights at each location.

For all the animals, morphological characteristics and body measurements were recorded for taxonomic identification. Rodent taxonomy was performed according to Patton and coworkers [24]. The bat identifications were confirmed following Gardner’s methodology [25]. Blood was collected from all the animals under anesthesia using 9:1 ketamine chlorhydrate (10%) and acepromazine (1%). All the small mammals, including the bats, that were used in these analyses received a collection number along with the initials of the collectors (YL and RM), and the animals were prepared for fluid preservation. These materials were subsequently deposited in the mammal collection at the Federal University of Espírito Santo (small non-volant mammals) and the Nacional Museum at the Federal University of Rio de Janeiro (bats).

### Dog survey

A search for dogs was conducted in houses near the locations where the wild mammals were captured. With the informed consent of their owners, blood samples were collected using Vacutainer^®^ tubes containing EDTA by puncturing each dog’s femoral vein. A questionnaire was used to record the age, sex, size, and primary function (hunting, companionship, or protection) of each dog. All dogs from the same house were considered to be a single event in this study.

### Parasitological survey

Parasitological and serological methods were used to identify *Trypanosoma* species in wild mammals and dogs. The parasitological methods included the examination of (i) fresh blood and (ii) hemocultures; for the latter, 0.3 to 0.6 ml of blood was inoculated into two tubes containing NNN/LIT medium for small mammals and dogs, one tube containing NNN/LIT for the isolation of *T*. *cruzi* and one tube containing NNN/Schneider’s medium for the isolation of trypanosomatids from bats.

The hemocultures were examined fortnightly for five months. Positive cultures, which demonstrated parasite growth, were amplified, cryopreserved, and deposited in the Coleção de *Trypanosoma* de Mamíferos Silvestres, Domésticos e Vetores, COLTRYP/Fiocruz. The sediments of the positive hemocultures that did not successfully amplify the parasites were centrifuged, and the pellets were stored at -20°C for the molecular characterization of the *Trypanosoma* species.

Serological analyses were performed only with the sera of non-volant wild mammals and dogs because no commercial anti-bat conjugate is available. For IgG antibody detection in the sera of wild mammals and dog, an indirect immunofluorescence antibody test (IFAT) assay was performed as described in [26]. Reference strains I00/BR/00F (TcI) and MHOM/BR/1957/Y (TcII) from axenic cultures were mixed in equal (1:1) proportions and used as antigens. The sera from Murinae rodents and plasma from dogs were tested with rat anti-IgG and dog anti-IgG, respectively, which were coupled with fluorescein isothiocyanate (Sigma, St. Louis, MO, USA). The sera of Echimyidae rodents and marsupials were tested as described in [20] using in-house anti-*Thrichomys* IgG and anti-*Didelphis* spp. IgG, respectively. The cut-off values for the IFAT were 1:40 for marsupials and dogs and 1:10 for rodents [27]. To confirm the dogs’ serological results, an enzyme-linked immunosorbent assay (ELISA) was performed. The cut-off value for the ELISA was the mean optical absorbance of the negative controls plus 20%. For the IFAT and ELISA, two negative and two positive control sera were added to each reaction. For the IFAT assays, specific positive and negative controls were added for each mammal order.

To exclude cross-reactions and to confirm mixed infections by *T*. *cruzi* and *Leishmania* sp., an IFAT using a mixture of axenic cultures containing *L*. *infantum* and *L*. *braziliensis* was performed. Mammals that presented higher serological titers for *Leishmania* sp. than for *T*. *cruzi* were considered to be infected by *Leishmania* sp. only when the *T*. *cruzi* titers were ≤1:80, and the presence of mixed infections was confirmed when both serological titers were >1:80 [28]. To test for cross-infection with *Leishmania* sp. in dogs, a rapid test for the diagnosis of canine visceral leishmaniasis (CVL) (TR DPP®, Bio-Manguinhos, FIOCRUZ, Rio de Janeiro, RJ, Brazil) was performed.

### Triatomine collection

After residents in rural locations in the Guarapari municipality reported the invasion of their residences by triatomines, health agents contacted them to discuss collection procedures and the delivery of the insects to the Zoonosis Control Center (ZCC). Between 2014 and 2015, the triatomines that were collected from distinct locations were delivered to and examined by our group (Fig 1).

The morphological identification of triatomines was performed according to [29], and the presence of flagellated *Trypanosoma* sp. forms in fecal material was observed by removing the intestinal content with scissors and forceps using optical microscopy. The intestinal content was diluted in phosphate-buffered saline (PBS) and stored at -20°C for *Trypanosoma* spp. characterization.

### Molecular characterization of cultures and intestinal content

The total genomic DNA from the mammalian blood cultures and triatomine intestinal contents was extracted using a phenol-chloroform method [30]. To identify infection of sylvatic mammals by *Trypanosoma* species, the DNA samples were subjected to a nested PCR for the small subunit (SSU) rRNA [11, 31] and gGAPDH [32] genes. For the identification of *Trypanosoma* sp. in triatomines, nested PCR was performed for only the SSU rRNA gene. All reactions included distilled water as a negative control. *Trypanosoma cruzi* strain SylvioX/10cl1 was used as a positive control.

The PCR products (~650 bp for the SSU rRNA gene and ~800 bp for the gGAPDH gene) were visualized using a 2% agarose gel stained with ethidium bromide and purified using an Illustra GFX PCR DNA and gel band purification kit (GE Healthcare Life Sciences, Little Chalfont, Buckinghamshire, UK). Both strands of DNA were then sequenced using a BigDye Terminator v3.1 Cycle Sequencing Kit (Applied Biosystems, Foster City, CA, USA) on an ABI 3730 DNA sequencer available at the PDTIS/Fiocruz sequencing platform.

The sequences were assembled and edited using SeqMan (DNASTAR Lasergene, Gatc, Konstanz, Germany) to obtain the SSU rRNA and gGAPDH consensus sequences, which were then aligned and corrected using BioEdit [33]. The sequences were compared to nucleotide sequences deposited in GenBank using the Basic Local Alignment Search Tool (BLAST) algorithm for initial screening. For the SSU rRNA and gGAPDH genes, phylogenies were inferred in Mega7 [34] using maximum likelihood (ML) tree inference under Kimura’s two-parameter model of nucleotide substitution with gamma-distributed variation among sites (K2P + G) for triatomines and non-volant mammals and a gamma-distributed rate with invariant sites (K2P + G + I) for bats. For the gGAPDH sequences, Tamura’s three-parameter model of substitution with invariant sites (T92P + I) was inferred for the *Monodelphis americana* isolate, and Tamura’s three-parameter model of nucleotide substitution with gamma-distributed variation among sites (T92 + G) was inferred for the bat isolates.

### SSU rRNA amplification and deep sequencing

The c624 isolate was subjected to another nested PCR of the SSU rRNA using the primers described above [11, 31]. For deep sequencing, the PCR products were single-end barcoded, purified using agarose gel electrophoresis (PureLink Quick Gel Extraction Kit, Invitrogen), quantified using a fluorometric assay (Qubit 2.0, Thermo Fisher Scientific) and pooled to equimolar concentrations for multiplexed, paired-end (2 × 300 bp) sequencing on an Illumina MiSeq platform (Reagent Kit v2) [23].

### Deep sequencing data analysis

Amplicon sequences were analyzed as described in [23]: after the sequence quality was verified in FastQC [35], the amplicons were filtered using windowed trimming in Sickle [36], retaining only full-length reads with ≥99.9% base call accuracy, which were then mapped against a *Trypanosoma* spp. reference collection from SILVA v119 [37] using Bowtie 2 [38]. Operational taxonomic unit (OTU) construction proceeded using the UPARSE algorithm in USEARCH [39] and BLAST-based taxonomic assignment in the QIIME environment [40], with run parameters established during prior *in silico* testing on trypanosomatid 18S rRNA sequences from NCBI. The samples were clustered into OTUs *de novo* at 98% sequence similarity and assigned to extant species with a confidence threshold of 80%.

After OTU establishment, the sequence read pairs for each OTU were merged and aligned in ClustalW (with the manual refinement of misplaced reads). Phylogenies were inferred in Mega7 [34] using ML tree construction under Kimura’s two-parameter model of nucleotide substitution with gamma-distributed variation among sites (K2P + G) and bootstrap values for 1000 replicates. The SSU rRNA and gGAPDH reference strains used for the Sanger and deep sequencing phylogenic analyses are listed with their accession numbers in S1 Table.

## Results

In this study, we observed substantial diversity among *Trypanosoma* species as well as among genotypes of *T*. *rangeli* and *T*. *c*. *cruzi* in a fragmented Atlantic Forest coastal area. Infection with distinct *Trypanosoma* species occurred primarily in bats, since only two non-volant wild mammal specimens from two species were infected, which constitutes an epizootic profile that is quite different from the profile that has usually been observed: the transmission cycle is occurring far from the residential areas.

### Non-volant sylvatic mammal occurrence and distribution in the Atlantic rainforest

During the four surveys, 73 small, non-volant sylvatic mammals were captured and classified into 12 species. The species richness was slightly higher in marsupials (seven species) than in rodents (five species) (Table 1). The species richness was similar among the three locations, but the species composition and relative abundances were distinct. We found nine species in Rio da Prata, eight species in Buenos Aires and six species in Amarelos (Table 1); we also accidentally captured two primates (*Callithrix geoffroyi*). The non-volant mammals’ relative abundances are presented in Table 1, which shows that the two most abundant species were *Metachirus nudicaudatus* (Didelphimorphia) and *Trinomys paratus* (Rodentia).

**Table 1:** Species richness (r) and relative abundance (%) of non-volant sylvatic mammals in Amarelos, Buenos Aires and Rio da Prata, Guarapari municipality, ES state, Brazil.

Species	Location			Total (r/%)
	Amarelos (r/%)	Buenos Aires (r/%)	Rio da Prata (r/%)	
Didelphis aurita*	4 (30.77)	1 (2.86)	1 (4.0)	6 (8.22)
*Gracilianus microtarsus*	-	1 (2.86)	-	1 (1.37)
*Marmosa paraguayana*	5 (38.46)	-	1 (4.0)	6 (8.22)
*Marmosa murina*	-	-	1 (4.0)	1 (1.37)
*Marmosops incanus*	1 (7.69)	3 (8.57)	1 (4.0)	5 (6.85)
Metachirus nudicaudatus*	-	13(37.15)	2 (8.0)	15 (20.55)
*Monodelphis americana*	-	2 (5.71)	-	2 (2.74)
*Akodon cursor*	1 (7.69)	4 (11.43)	7 (28.0)	12 (16.44)
*Necromys lasiurus*	-	-	1 (4.0)	1 (1.37)
*Nectomys squamipes*	1 (7.69)	2 (5.71)	5 (20)	8 (10.95)
*Rhipidomys mastacalis*	1 (7.69)	-	-	1 (1.37)
Trinomys paratus*	-	9 (25.71)	6 (24)	15 (20.55)
Total	13 (17.81)	35 (47.94)	25 (34.25)	73 (100)

r/%: species richness/relative abundance

*The star represents mammals that presented positive results on the serological exam.

### Occurrence and distribution of bats

One hundred eighty-six bat specimens from 17 distinct species were examined during the four sampling events. Only seven bat species were common to the three study sites (Table 2). Moreover, the species richness differed among the three locations; Amarelos presented the highest bat species richness, and Buenos Aires presented the lowest (Table 2). *Artibeus lituratus* and *Carollia perspicillata* were the most abundant bat species. The Amarelos location presented the highest number of captured bats, and *A*. *lituratus*, *C*. *perspicillata*, and *Desmodus rotundus* were the most abundant. At the Buenos Aires location, the primary species captured were *A*. *lituratus*, *C*. *perspicillata*, and *Sturnira lilium*. At the Rio da Prata location, *Anoura geoffroyi*, *C*. *perspicillata*, and *Rhinophylla pumilio* were the most abundant species (Table 2).

**Table 2:** Species richness and relative abundance of bats (%) in Amarelos, Buenos Aires and Rio da Prata, Guarapari municipality, ES state, Brazil.

Species	Location			Total (r/%)
	Amarelos (r/%)	Buenos Aires (r/%)	Rio da Prata (r/%)	
*Anoura caudifer*	3 (3.75)	-	6 (8.95)	9 (4.84)
*Anoura geoffroyi*	-	-	9 (13.43)	9 (4.84)
*Artibeus fimbriatus*	1 (1.25)	2 (5.13)	1 (1.49)	4 (2.15)
*Artibeus lituratus*	15 (18.75)	9 (23.08)	5 (7.46)	29 (15.59)
*Carollia perspicillata*	30 (37.5)	14 (35.90)	25 (37.31)	69 (37.10)
*Desmodus rotundus*	9 (11.25)	-	1 (1.49)	10 (5.38)
*Glossophaga soricina*	4 (5.00)	2 (5.13)	-	6 (3.22)
*Micronycteris* sp.	2 (2.50)	-	-	2 (1.07)
*Myotis nigricans*	2 (2.50)	1 (2.56)	1 (1.49)	4 (2.15)
*Phyllostomus discolor*	2 (2.50)	1 (2.56)	2 (2.98)	5 (2.69)
*Phyllostomus hastatus*	3 (3.75)	2 (5.13)	-	5 (2.69)
*Platyrrhinus lineatus*	-	-	4 (5.97)	4 (2.15)
*Platyrrhinus recifinus*	1(1.25)	1 (2.56)	3 (4.48)	5 (2.69)
*Rhinophylla pumilio*	3 (3.75)	-	7 (10.45)	10 (5.38)
*Sturnira lilium*	3 (3.75)	7 (17.95)	3 (4.48)	13 (6.98)
*Tonatia bidens*	1 (1.25)	-	-	1 (0.54)
*Trachops cirrhosus*	1 (1.25)	-	-	1 (0.54)
Total	80 (43.01)	39 (20.97)	67 (36.02)	186 (100)

r/%: species richness/relative abundance

### *Trypanosoma* spp. infection in sylvatic non-volant mammals and bats

**Fig 2:**
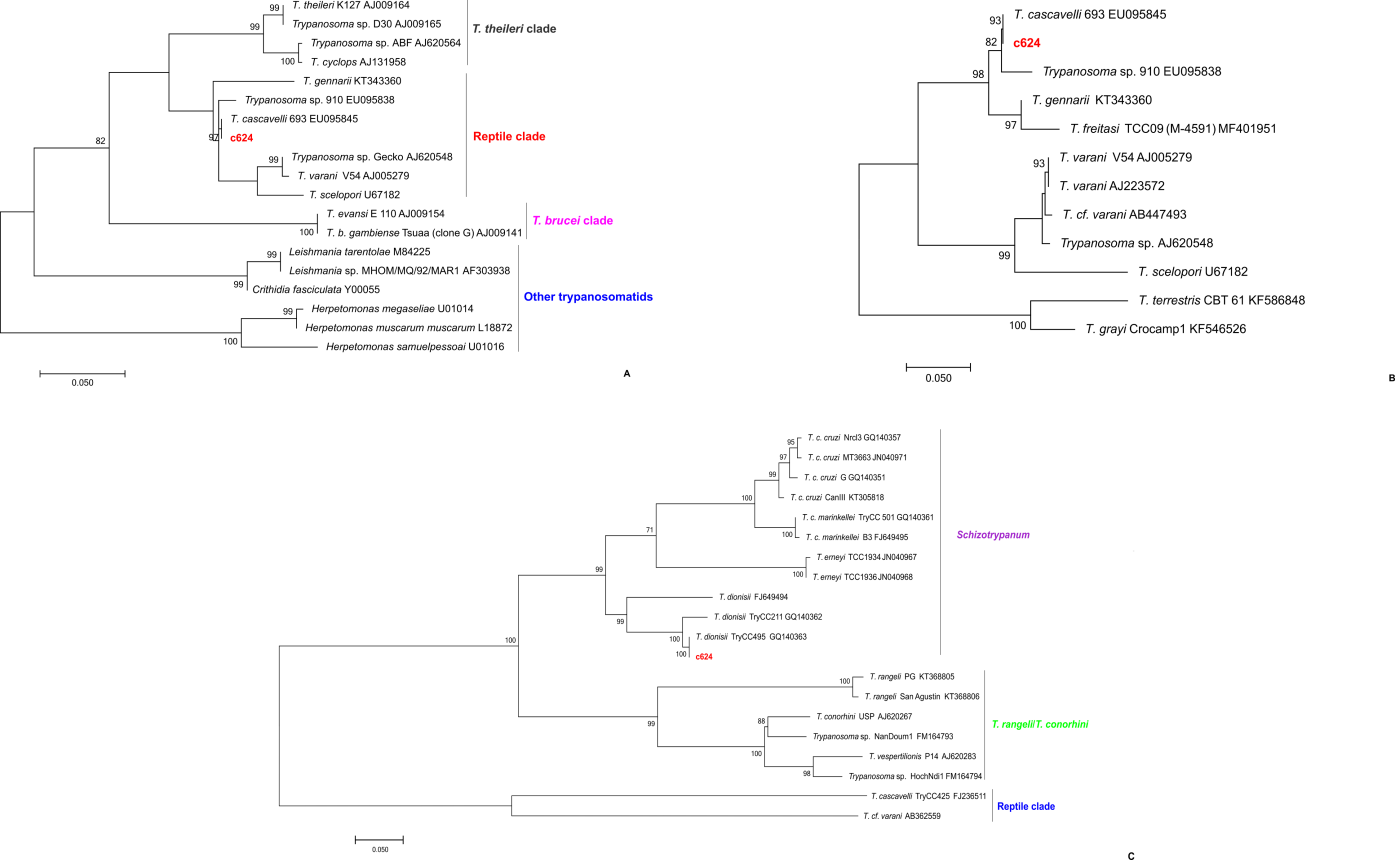
Phylogenetic placements of SSU rRNA and gGAPDH sequences from hemocultures of *Monodelphis americana*. The tree was inferred by maximum likelihood using the Kimura 2-parameter model with a gamma-distributed rate of variation among sites (K2P + G) for SSU rRNA and the Tamura 3-parameter model of substitution with invariant sites (T92P + I) for gGAPDH. The numbers at the nodes indicate support from 1000 bootstrap pseudoreplicates. (A) SSU rRNA showed the c624 isolate clustered in the *Trypanosoma* reptile clade; (B) based on SSU rRNA, the c624 isolate was identified as *T*. *cascavelli*; (C) based on gGAPDH, the c624 isolate was identified as *T*. *dionisii*.

**Fig 3:**
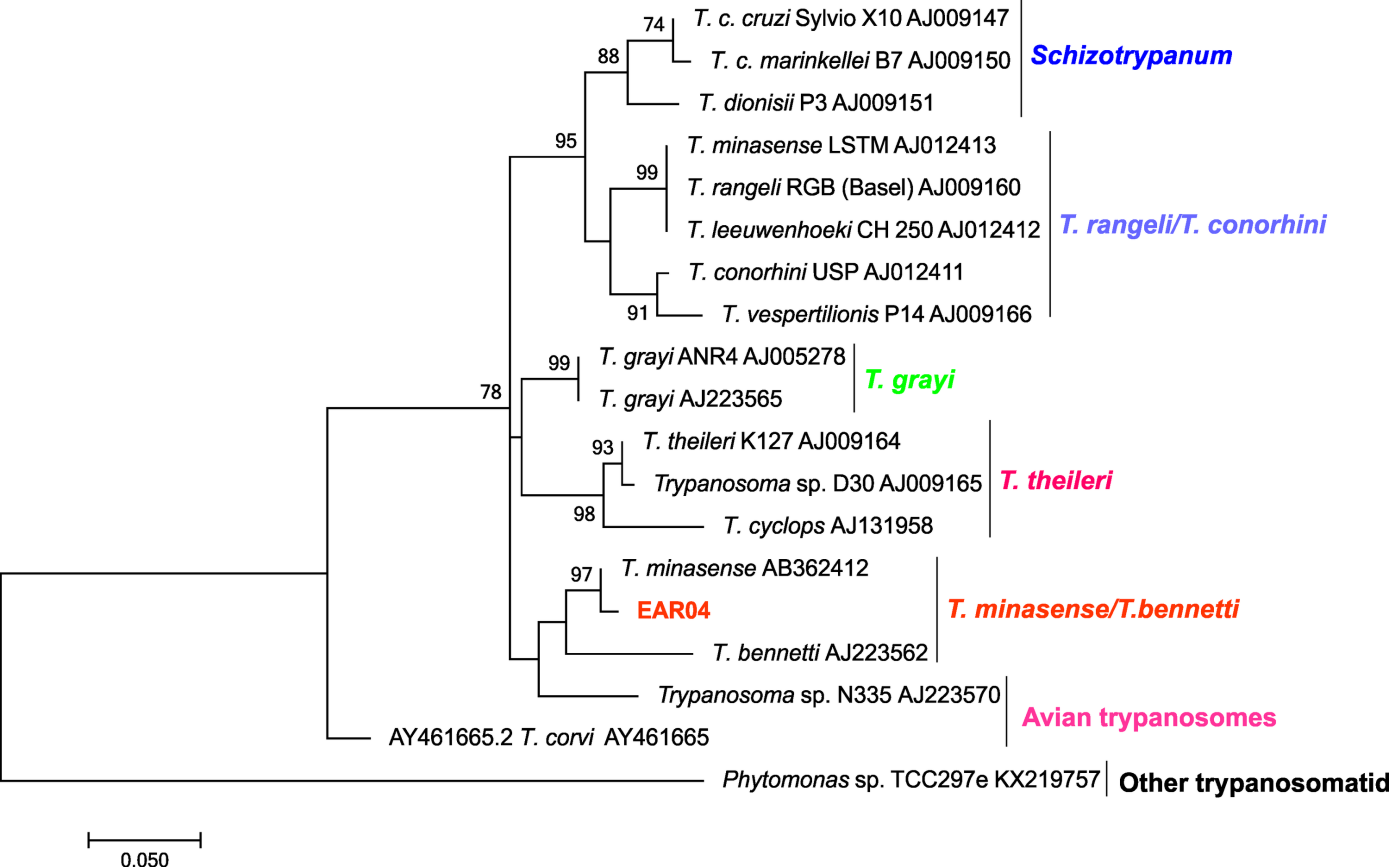
Phylogenetic placement of SSU rRNA sequences from *Callithrix geoffroyi* hemocultures. The tree was inferred by maximum likelihood using the Kimura 2-parameter model plus a gamma-distributed rate of variation among sites (K2P + G). The numbers at the nodes indicate support from 1000 bootstrap pseudoreplicates. The sample clustered with *T*. *minasense* from the red-handed tamarin in the same branch as *T*. *bennettii*.

The prevalence of *Trypanosoma* spp. infection was higher in bats (22.66%) than in sylvatic non-volant wild mammals (2.67%). Only two non-volant mammal specimens of two species were found to be infected with *Trypanosoma* species, as demonstrated by positive hemocultures, and they were *M*. *americana* (2851-c624) in Buenos Aires and *C*. *geoffroyi* (EAR04) in Amarelos. The SSU rRNA marker was amplified in both samples, and, to our surprise, the ML tree showed the presence of a *Trypanosoma* sp. from a reptile clade that clustered with *T*. *cascavelli* (Fig 2A, Fig 2B, Table 3) in the *M*. *americana* isolate, and the gGAPDH marker showed that this marsupial specimen was also infected with *T*. *dionisii* (Fig 2C). The EAR04 sample clustered with *T*. *minasense* (Fig 3, Table 3). Serologically, two marsupial specimens, one *Didelphis aurita* (1:320) and one *M*. *nudicaudatus* (1:80) from Buenos Aires, and two *T*. *paratus* specimens, one from Buenos Aires (1:40) and one from Rio da Prata (1:20), were positive for *T*. *cruzi* infection (Table 1).

**Table 3:** *Trypanosoma* spp. identification using SSU rRNA and gGAPDH in sylvatic mammals captured in the Guarapari municipality, ES state, Brazil.

Species	ID	Location	SSU rRNA	gGAPDH
*Anoura* spp.	c596	Rio da Prata	*T*. *dionisii*	*T*. *dionisii*
	c621, c621s			
*Artibeus* spp.	c700	Buenos Aires	*T*. *cruzi* TcI	NA
*Artibeu* spp.	RM 837	Amarelos	*T*. *cruzi* TcIII/V	NA
*C*. *geoffroyi*	EAR04	Amarelos	*T*. *minasense*	NA
*C*. *perspicillata*	c593	Rio da Prata	*T*. *rangeli* D	*T*. *rangeli* D
	c594	Amarelos	*T*. *dionisii*	*T*. *dionisii*
	c595	Rio da Prata	*T*. *dionisii*	*T*. *dionisii*
	c597	Buenos Aires	*T*. *dionisii*	*T*. *dionisii*
	c598	Rio da Prata	*T*. *dionisii*	*T*. *dionisii*
	c622, c622s	Amarelos	*T*. *dionisii*	*T*. *dionisii*
	c623	Buenos Aires	*T*. *dionisii*	*T*. *dionisii*
	c625	Rio da Prata	*T*. *dionisii*	*T*. *dionisii*
	c626	Amarelos	*T*. *dionisii*	*T*. *dionisii*
	c681	Rio da Prata	*T*. *dionisii*	NA
	c688	Amarelos	*T*. *dionisii*	NA
	c692	Amarelos	*T*. *dionisii*	*T*. *c*. *cruzi* TcI
	RM851	Rio da Prata	*T*. *cruzi* TcIII/V	NA
	RM2028	Amarelos	*T*. *rangeli* B	*T*. *rangeli* B
	RM 2054	Buenos Aires	*Trypanosoma* sp.	*Trypanosoma* sp.
*D*. *rotundus*	c694	Amarelos	*T*. *cruzi* TcI	*T*. *cruzi* TcI
	RM823		*T*. *cruzi* TcIII/V	*Trypanosoma* sp.
	RM2027		*Trypanosoma* sp.	NA
*G*. *soricina*	c620	Amarelos	*T*. *dionisii*	*T*. *dionisii*
*M*. *americana*	c624	Buenos Aires	*T*. *cascavelli*	*T*. *dionisii*
*M*. *nigricans*	RM838	Amarelos	*T*. *c*. *cruzi* TcIII/V	*Trypanosoma* sp.
*P*. *discolor*	RM 742	Buenos Aires	*T*. *c*. *marinkellei*	*T*. *c*. *marinkellei*
	RM 842	Rio da Prata	*T*. *c*. *marinkellei*	NA

NA: Not amplified due low DNA quantities.

**Fig 4:**
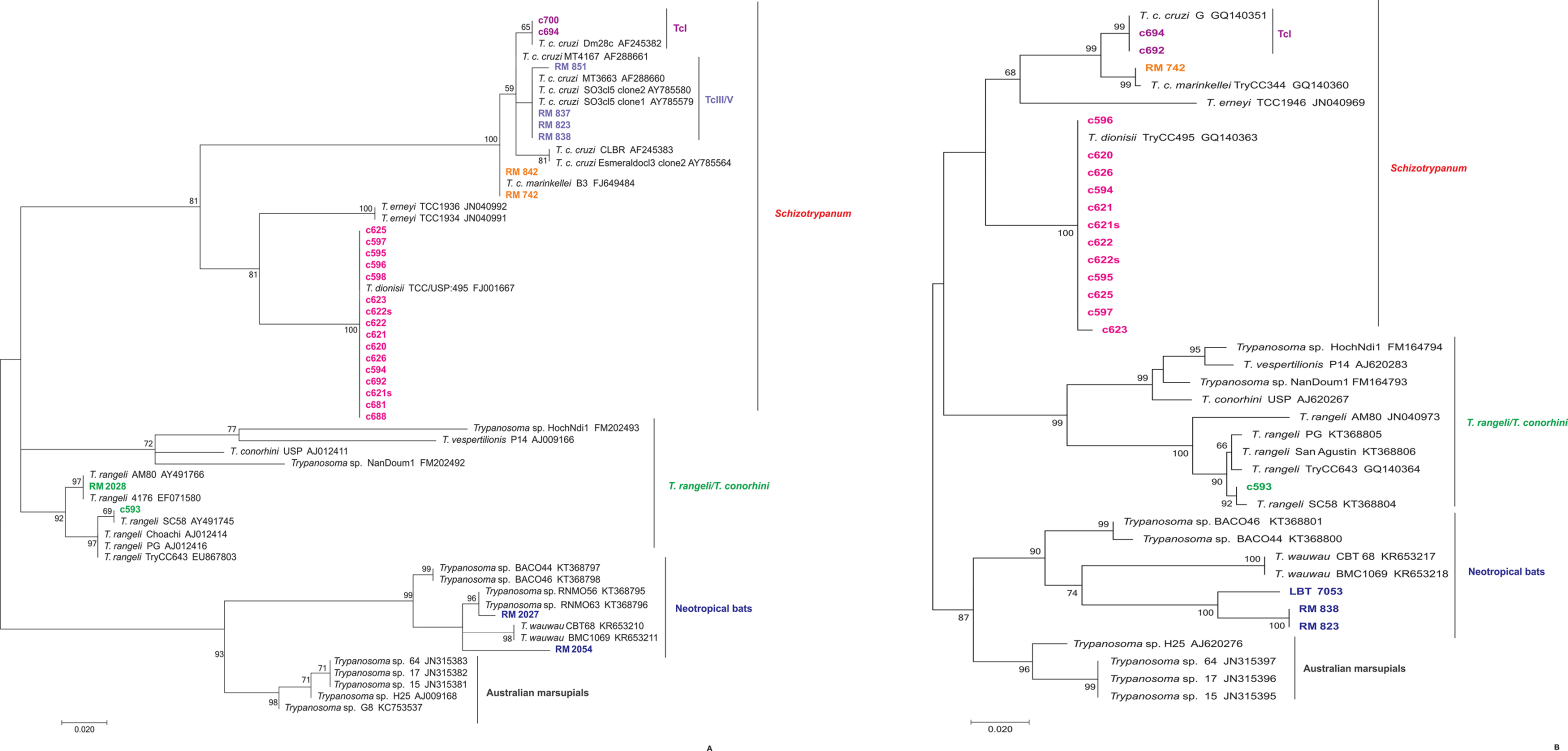
Phylogenetic placements of SSU rRNA and gGAPDH sequences from bat hemocultures. The tree was inferred by maximum likelihood using the Kimura 2-parameter model with a gamma-distributed rate with invariant sites (K2P + G + I) for SSU rRNA and the Tamura 3-parameter model and gamma-distributed variation among sites (T92P + G) for gGAPDH. The numbers at the nodes indicate support from 1000 bootstrap pseudoreplicates. (A) The samples clustered within the *T*. *cruzi* clade, in the *Schizotrypanum* group (*T*. *c*. *cruzi*, *T*. *c*. *marinkellei*, and *T*. *dionisii*), in the *T*. *rangeli*/*conorhini* group (*T*. *rangeli*), and with *Trypanosoma* species from Neotropical bats. Two *T*. *c*. *cruzi* genotypes and two *T*. *rangeli* lineages were identified as TcI, TcIII/V, and lineages B and D, respectively. (B) Samples RM 823, RM 838, and LBT 7053 clustered with *Trypanosoma* species from Neotropical bats. Sample c692 clustered within the *Schizotrypanum* group (*T*. *c*. *cruzi*).

Forty-four bats from eight genera/species presented positive fresh blood smears or hemocultures. Amarelos had the highest number of bats infected with *Trypanosoma* species, with an infection rate of 45.45%. The infection rate among bats from Rio da Prata was 38.63%, and the infection rate among bats from Buenos Aires was 15.92%. *Carollia perspicillata* was the primary bat species presenting the highest number of infected bat specimens (Fig 4).

**Fig 5:**
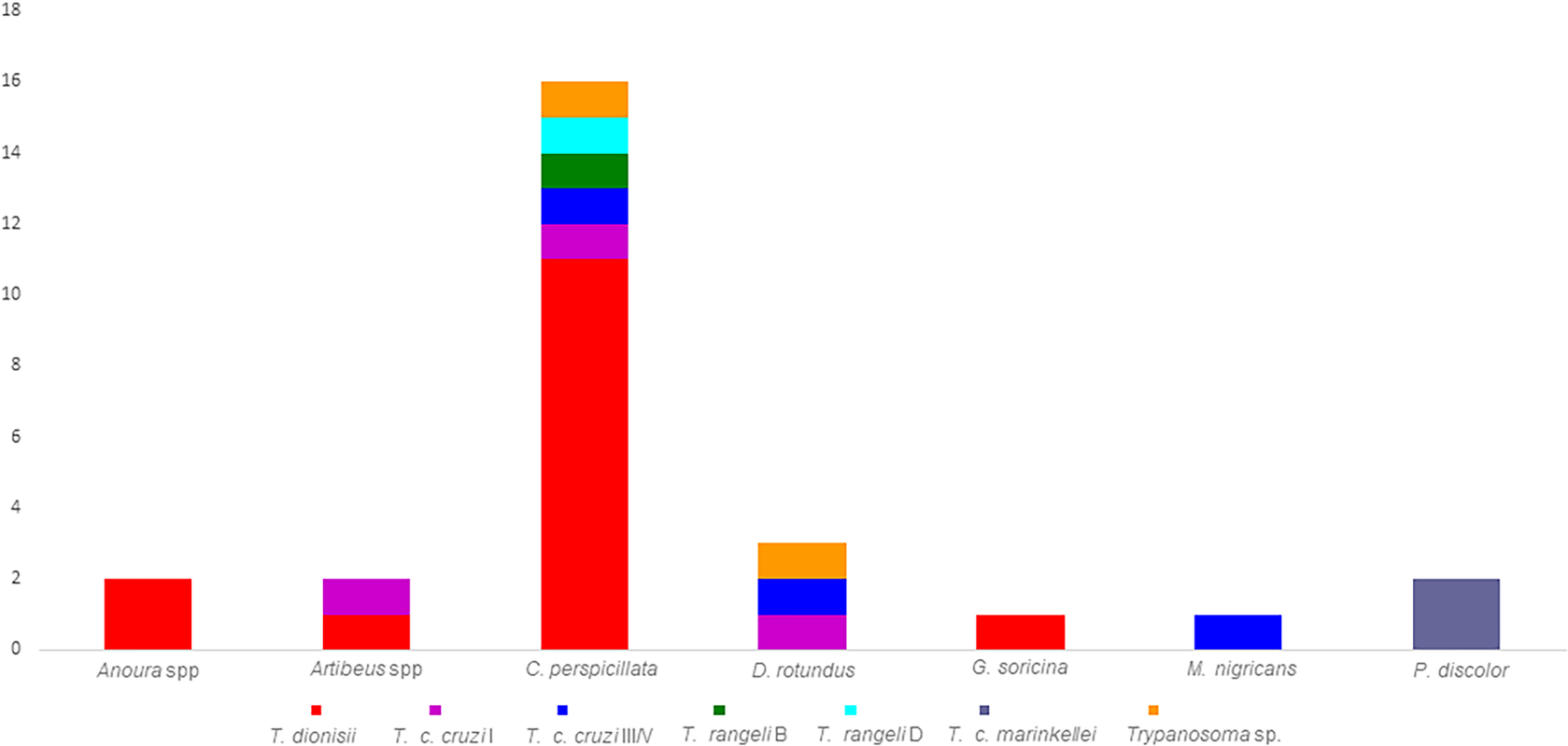
*Trypanosoma* spp. infection in bats captured in Guarapari municipality, ES state, Brazil. The column represents the *Trypanosoma* infection profile of each bat species. The colors represent each *Trypanosoma* species identified in bats.

The trypanosome isolates from 26 bats were characterized using SSU rRNA, and 19 isolates were characterized using gGAPDH sequencing to identify the *Trypanosoma* species circulating in the three study locations. Three *Trypanosoma* species were identified, *T*. *cruzi*, *T*. *dionisii*, and *T*. *rangeli*, in addition to a not-yet-described *Trypanosoma* sp. from Neotropical bats (Fig 4A, Fig 4B). *Trypanosoma dionisii* was the most predominant species (56%) among bats (Fig 5) and was identified in bats from the three locations together with *T*. *c*. *cruzi*. *Trypanosoma cruzi marinkellei* was identified in bats collected in Buenos Aires and Rio da Prata sites. *Trypanosoma rangeli* lineages B and D were found to infect bats at the Amarelos and Rio da Prata sites, and a *Trypanosoma* sp. similar to a species from Neotropical bats was observed in Buenos Aires and Amarelos (Table 3). *Carollia perspicillata* specimens had the highest number of *Trypanosoma* species, but this bat species was not found to be infected by *T*. *c*. *marinkellei* (Table 3). The gGAPDH and SSU rRNA results differed in terms of the *Trypanosoma* spp. identified in three samples (Fig 4B, Table 3). Two samples identified as *T*. *c*. *cruzi* TcIII/V based on SSU rRNA were classified as *Trypanosoma* sp. from Neotropical bats, and one sample identified as *T*. *dionisii* based on SSU rRNA was classified as *T*. *c*. *cruzi* TcI; we confirmed that these samples had mixed infections by *T*. *c*. *cruzi* TcIII/V-*Trypanosoma* sp. and *T*. *dionisii*-*T c*. *cruzi* TcI. The sample LBT 7053 was also confirmed to be infected by *Trypanosoma* sp. from Neotropical bats. DNA from the hemocultures of eight bat samples did not amplify, and the sequences from sample c683 were ambiguous, likely due to a mixed infection.

### *Trypanosoma* spp. identification by deep sequencing

**Fig 6:**
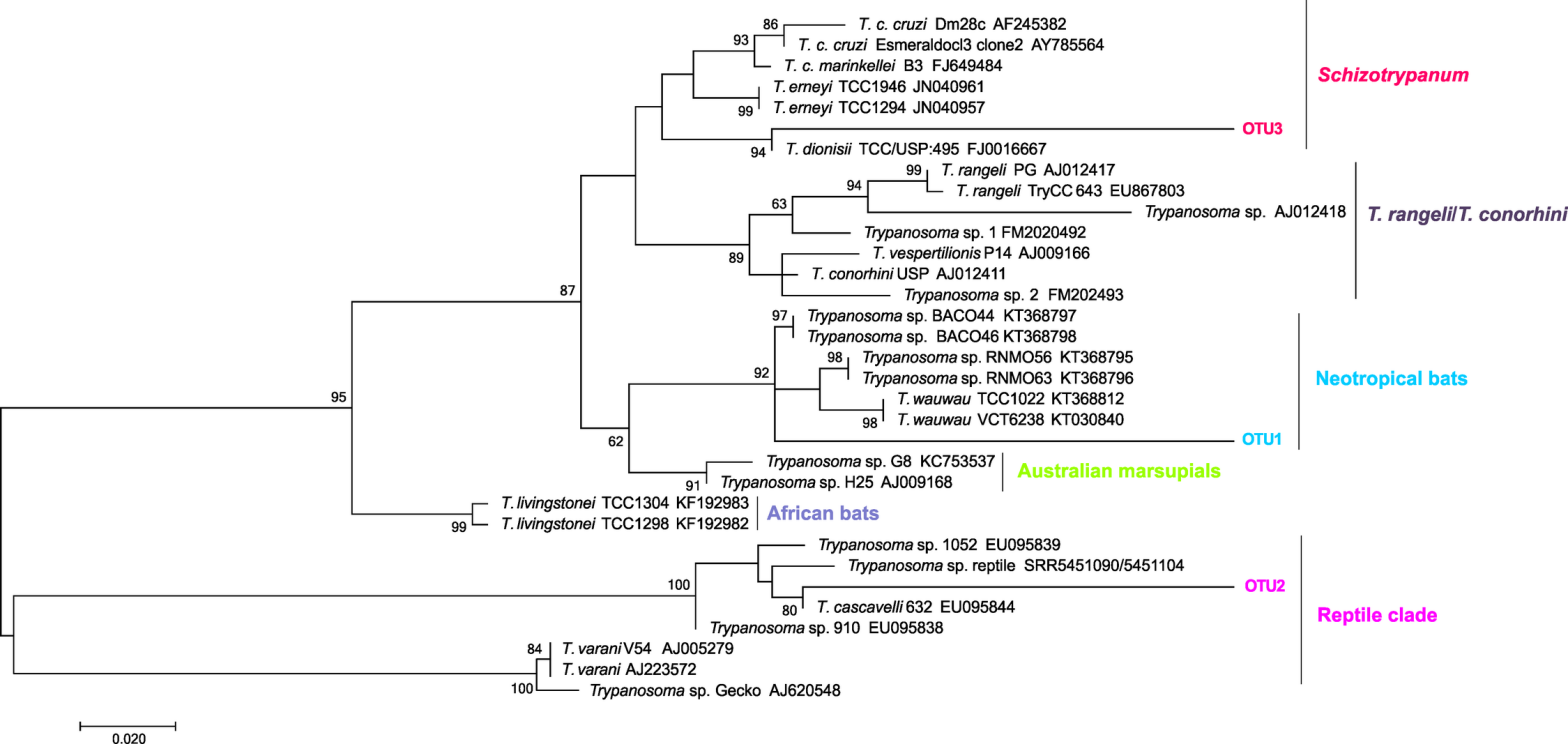
Phylogenetic placement of *Trypanosoma* OTUs detected in *Monodelphis americana* from the Guarapari municipality, ES state, Brazil. The tree was constructed based on 18S rRNA using the maximum likelihood method with Kimura’s 2-parameter model and gamma-distributed variation among sites (K2P + G). The numbers at the nodes indicate support from 1000 bootstrap pseudoreplicates. Two OTUs clustered within the *T*. *cruzi* clade (OTUs 1 and 3), and one OTU clustered within the reptile clade (OTU 2).

Based on deep sequencing, *M*. *americana* isolate c624 exhibited a mixed infection with three OTUs: *Trypanosoma* sp. (OTU 1), *T*. *cascavelli* (OTU 2) and *T*. *dionisii* (OTU 3). In the phylogenetic analysis, OTU 1 clustered with the *Trypanosoma* species from Neotropical bats (*T*. *wauwau* and *Trypanosoma* sp. RNMO and BACO); OTU 2 clustered within the reptile clade in the same branch as *T*. *cascavelli* 632; and OTU 3 clustered within the *Schizotrypanum* subgenus in the same branch as *T*. *dionisii* TCC/USP:495 (Fig 6).

### *Trypanosoma cruzi* survey in dogs

Fifty-five dogs were examined during the four surveys, 18 from Amarelos, 17 from Buenos Aires and 20 from Rio da Prata. Among the serological tests performed on the dog samples, nine presented borderline titers (serological titers = 1:40), and four dogs from Amarelos, two from Buenos Aires and one from Rio da Prata presented positive titers for *T*. *cruzi* (Table 4). None of the dogs presented positive fresh blood smears or hemocultures.

**Table 4:** Serological survey of dogs in Amarelos, Buenos Aires and Rio da Prata, Guarapari municipality, ES state, Brazil.

Location	IFAT	ELISA
Amarelos (n = 6)	1:40 (n = 2); 1:80 (n = 2); 1:160 (n = 1); 1:320 (n = 1)	Positive
Buenos Aires (n = 4)	1:40 (n = 2); 1:80 (n = 2)	Positive
Rio da Prata (n = 6)	1:40 (n = 5); 1:80 (n = 1)	Positive

### *Trypanosoma* spp. infection in triatomines

We received 79 adult triatomine specimens between 2014 and 2015 from different rural areas in the Guarapari municipality (Fig 1). Seventy-three specimens were identified as *T*. *vitticeps* (92.40%), and six were identified as *P*. *geniculatus* (7.60%). The *Trypanosoma* infection rates observed via the intestinal content examinations with optical microscopy were high for both: 52% in the former and 50% in the latter.

**Fig 7:**
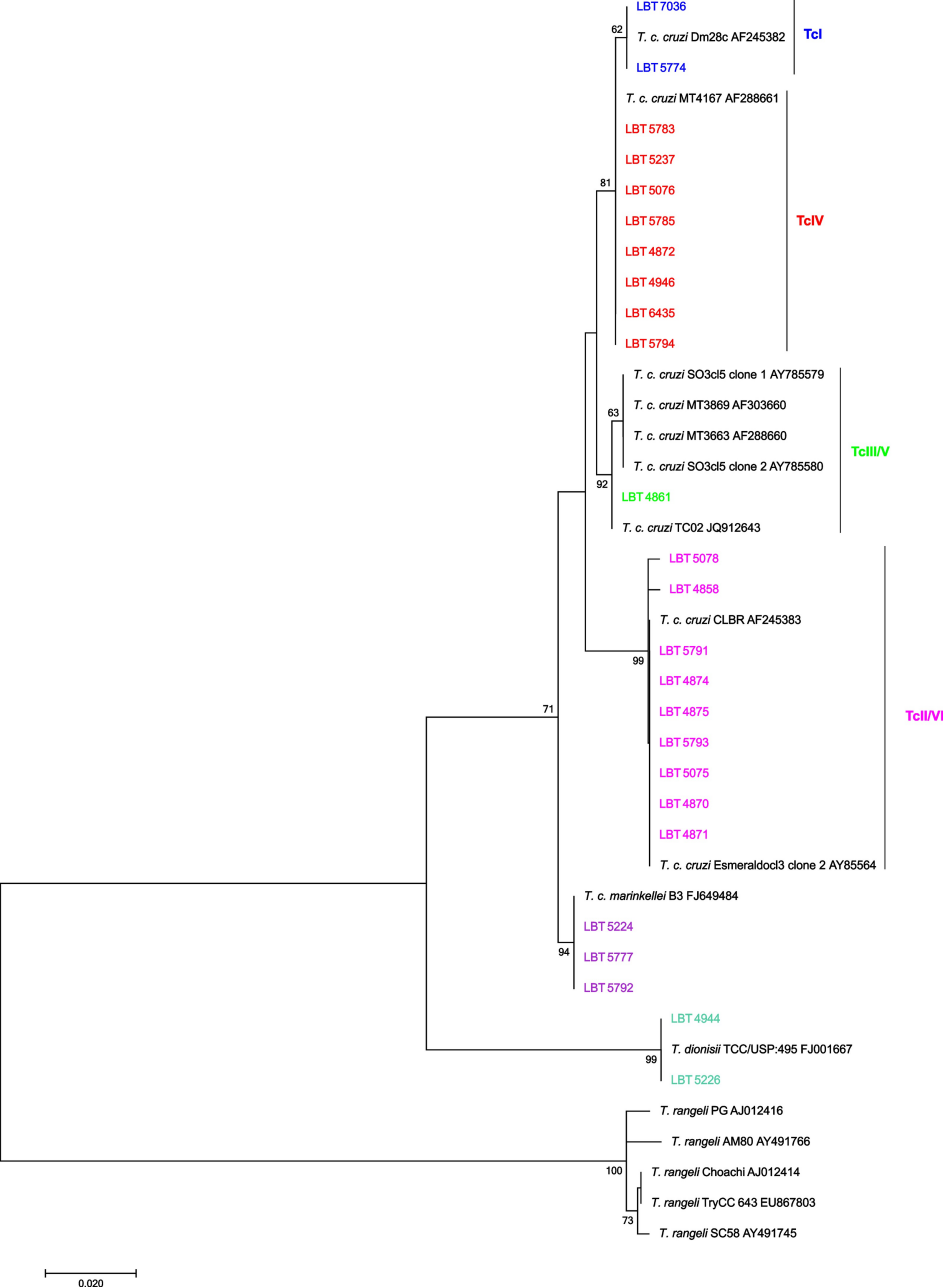
Phylogenetic placement of SSU rRNA sequences from the intestinal contents of *Triatoma vitticeps*. The tree was inferred by maximum likelihood using the Kimura 2-parameter model plus a gamma-distributed rate of variation among sites (K2P + G). The numbers at the nodes indicate support from 1000 bootstrap pseudoreplicates. The samples were clustered into three distinct groups: *T*. *c*. *cruzi*, *T*. *c*. *marinkellei* and *T*. *dionisii*. Four *T*. *c*. *cruzi* DTUs were identified: TcI, TcII, TcIII/V and TcIV.

Forty-seven DNA samples were extracted to directly identify *Trypanosoma* species from the intestinal content: 37 from positive samples and eight from negative samples, which were randomly selected. DNA from five samples, four from *T*. *vitticeps* and one from *P*. *geniculatus*, could not be extracted. Forty-two samples were PCR-positive (~650 bp), and they were sequenced. Seven samples that were negative based on the examination of intestinal content using optical microscopy were positive according to the PCR analysis, increasing the *Trypanosoma* species infection rates. Four samples that were positive for *Trypanosoma* sp. based on optical microscopy did not amplify in the PCR. Twenty-five samples (59.52%) had single infections, and 17 samples (40.48%) had sequences with ambiguities, likely due to mixed infections. Phylogenetic analysis (Fig 7) revealed that *T*. *c*. *cruzi* was the predominant species. We observed four circulating DTUs: TcI, TcII, TcIII/V and TcIV. TcII was the most prevalent DTU (Table 5). We found *T*. *vitticeps* specimens infected with *T*. *c*. *marinkellei* and *T*. *dionisii* (Table 5).

**Table 5:** *Trypanosoma* ssp. occurrence and identification in *Triatoma vitticeps* collected from distinct locations in Guarapari municipality, ES state, Brazil.

Species	DTU	Number of samples
*Trypanosoma cruzi cruzi*	TcI	01
	TcII	09
	TcIII/V	01
	TcIV	07
*Trypanosoma cruzi marinkellei*	NA	03
*Trypanosoma dionisii*	NA	02

NA: not applicable

## Discussion

The epizootic scenario observed in 2012 in the area of a fatal aCD case in Guarapari municipality, ES state [22], was currently the same approximately two years later, showing that these *Trypanosoma* spp. transmission cycles are stable and well-established in the study region. A reservoir is defined as a system formed by a group of species capable of maintaining a certain parasite in nature [41]. In this study, although we focused on reasonably preserved areas and used a higher number of captured mammals than in the previous study [22], we found only bats infected with *T*. *cruzi*. Thus, we can confirm that bats are the reservoir system in this Atlantic Forest area.

We observed a diversity of bat species at the three study sites, demonstrating that environmental changes in the examined Atlantic rainforest fragment did not drastically impoverish the local bat biodiversity. In terms of bat identification, we found a fairly representative number of species. Of the 42 species of Phyllostomidae and ten species of Vespertilionidae that have already been described in ES state [42], we found 38% and 10%, respectively. We observed that environmental variables interfered with the distributions of the bats, as only 1/3 of the bat species were found at the three study sites. The predominance of phyllostomids, especially the generalists *C*. *perspicillata* and *A*. *lituratus*, was expected because Phyllostomidae is the most common family in the Neotropical region [43].

Our data confirm that bats are suitable reservoir hosts for several *T*. *c*. *cruzi* DTUs as well as *Trypanosoma* species of the *T*. *cruzi* clade, which may occur in single or mixed infections. Because of the diversity of the *T*. *cruzi* clade observed among bats captured in the Atlantic Forest, these data support the bat seeding hypothesis. We also confirm that bats are the primary reservoir hosts of *T*. *c*. *cruzi* in this area and have increased our knowledge regarding the range of bat species that harbor *T*. *c*. *cruzi* genotypes, having observed TcI and TcIII/V infections in *Artibeus* spp., *D*. *rotundus* and *M*. *nigricans*. Dario and coworkers [23] previously observed TcI and TcIII/V in *Anoura* spp., *C*. *perspicillata*, and *R*. *pumilio*. Here, we once again observed *T*. *c*. *cruzi* infecting the generalist *C*. *perspicillata* as well as the hematophagous bat species *D*. *rotundus*. Additionally, the presence of *T*. *dionisii* and *T*. *c*. *marinkellei* has already been reported in Atlantic rainforest in northern ES state. Our results show that the occurrence of *T*. *c*. *marinkellei* in the Atlantic rainforest biome is not occasional and that its occurrence is not restricted to the Amazon and Pantanal regions [44–45].

We observed a broader lineage and host species distribution for *T*. *rangeli*, since only lineages A and E have been previously reported in bats [46]. *Trypanosoma rangeli* lineage B has been described as being exclusive to the Amazon region, infecting primates and humans [47–49]. In addition, this parasite has been transmitted to *Rhodnius* species [47–48, 50]. Here, we confirmed that other triatomine species are responsible for its transmission, as other *Rhodnius* spp. have not been reported in this area. Other authors, including Steindel and coworkers [51], have reported the isolation of *T*. *rangeli* from *P*. *megistus* in Santa Catarina state.

Bat trypanosomes are morphologically identical, as observed in the *T*. *cruzi* clade in the subgenus *Schizotrypanum*, species that were all considered *T*. *cruzi*-like in the past [3]. Perhaps for these reasons, bat trypanosomatids still represent an undiscovered world. The use of powerful analytical methodologies, such as molecular tools with high discriminatory power, has enabled the identification of several new trypanosome species. Recently, many research groups worldwide have reported new *Trypanosoma* species that infect bats and are associated with the *T*. *cruzi* clade [7, 10, 14, 52], increasing the likelihood of understanding the role played by bats in the origin, diversity, and ecology of trypanosomatids. We encountered two trypanosome samples that clustered into the Neotropical bat groups, with one in the same branch as a *Trypanosoma* sp. from Neotropical bats [10] and the other likely a new species. These trypanosomes from Neotropical bats have been reported in Brazil, Bolivia, Ecuador and Panama [10, 53–55]. Thus, by detecting *Trypanosoma* spp. in *C*. *perspicillata*, *D*. *rotundus*, and *M*. *nigricans* (a bat species from the Vespertilionidae family), in the Atlantic rainforest, we have extended the host range of *Trypanosoma* spp. in Neotropical bats.

Bats are known to host different trypanosome species and have been suggested to be the ancestral hosts of the *T*. *cruzi* clade [15]. A high trypanosome infection rate was observed in the study areas for *C*. *perspicillata*, which was the primary captured/analyzed species. In addition, we found a lower species diversity due to the isolation method (hemoculture) that was used. The ability of bats to host so many distinct species could be explained by their diverse behavior, which could facilitate the transmission/dispersal of trypanosomes. Bats are generalists in terms of their feeding habits, which include the consumption of insects, and they have a long lifespan [56–57], which may increase their chances of acquiring trypanosome infections. In addition, some chiropteran species are capable of living in small or large colonies [58–61], and they habitually groom and regurgitate for one another [62–64]. These behavioral traits may enhance *Trypanosoma* spp. transmission.

*Triatoma vitticeps*, the primary vector found in this Atlantic rainforest area, was observed to maintain single and mixed infections with four *T*. *c*. *cruzi* DTUs. The same DTUs were described in a human infection in the same area [22]. *Trypanosoma cruzi cruzi* TcI is considered the most frequent *T*. *c*. *cruzi* DTU circulating on the American continent [65], but it was not observed frequently in the study area, where the predominant type was TcII. The predominance of TcII corroborates the notion that this DTU is maintained successfully by several host species, including bats, in the sylvatic environment of the Atlantic rainforest, as has already been shown among other wild mammal taxa [66–67].

We found six *T*. *vitticeps* specimens infected by *T*. *c*. *marinkellei* and *T*. *dionisii*, and this is the first time these species have been observed in the *Triatoma* genus. *Trypanosoma cruzi marinkellei* is known to be transmitted by triatomines of the *Cavernicola* genus, and *Rhodnius* spp. have been infected experimentally, as shown by xenodiagnosis [68–69]. This study is the first report of *T*. *dionisii* infection in triatomines. *Trypanosoma dionisii* transmission is associated with cimicid bugs [70], but there have been no reports of this bug taxon in ES state. Importantly, because they are members of the same subgenus (*Schizotrypanum*), *T*. *c*. *cruzi* and *T*. *dionisii* can likely share the same invertebrate hosts, and infection of cimicid bugs by *T*. *c*. *cruzi* has previously been reported [71]. We do not know whether it is possible for *T*. *dionisii* to be transmitted to mammalian hosts through vectorial contamination, but human infections by *T*. *dionisii* via the oral route have already been described [22].

In our study, we observed 12 species of small, sylvatic non-volant mammals (rodents and marsupials) in three Guarapari Atlantic rainforest fragments; this figure corresponds to 25.5% of the marsupial and rodent species that have been described in ES state [72] and is therefore a reflection of an environmental disturbance. We observed *D*. *aurita* in all of the sites, but it was not the most abundant species in any of the areas. Some species, such as *M*. *americana*, *G*. *microtarsus*, *R*. *mastacalis*, and *T*. *paratus*, which were recorded in the Buenos Aires location, are typical of less disturbed environments, indicating that this is a reasonably preserved area.

One *M*. *americana* specimen presented mixed infection by *T*. *cascavelli*, *T*. *dionisii* and *Trypanosoma* sp. The observation of *T*. *dionisii* infecting a marsupial reinforces that this parasite is not restricted to infecting only bats. *Trypanosoma dionisii* has already found in a human infection [22], but the unexpected finding of *T*. *cascavelli* infections in mammals is intriguing, as this species was described in *Crotalus durissus*, a species of snake [73–75]. The current study is not the first to find trypanosomes from the reptile clade infecting sylvatic mammals, as this occurrence has already been observed in *C*. *perspicillata* and *D*. *rotundus* bats [23]. Little is known about how *T*. *cascavelli* is maintained in nature. Sand flies have been hypothesized to be involved in its transmission cycle, given that the transmission of anuran and reptilian trypanosomes by sandflies has previously been described [76–79], and this trypanosome has been isolated from these insects [74]. The infection of mammals by this trypanosome could also be occurring via sandflies in this area.

Analysis of this situation raises questions regarding the ancestral and secondary hosts of *T*. *cascavelli* in nature. We can hypothesize that marsupials are the ancestral hosts of this trypanosome species and that snakes are accidental hosts. *Monodelphis americana* presents insectivorous-omnivorous feeding habits [80] and might contract infections by *T*. *cascavelli* via the oral route (predation of insects). Therefore, snakes could be infected through their predation of small mammals, including small marsupials. Marsupials have a lower body temperature than placental mammals [81], and this condition could have facilitated the adaptation of this trypanosome to cold-blooded animals. In fact, mammals from the *Monodelphis* genus have low body temperatures, between 32 and 34°C, and they can attain much lower body temperatures [82–83]. In addition, other trypanosome species infecting members of the lizard/snake clade have been isolated from marsupials. *Trypanosoma gennarii* [84] was isolated from the marsupial *M*. *domestica* in the Cerrado biome, while *T*. *freitasi* was isolated for the first time in 1957 from *Didelphis albiventris* and later from *D*. *marsupialis* [85–86]. The hypothesis that marsupials were the first hosts seems to be the most parsimonious, since the converse, i.e., snakes being the original hosts of *T*. *cascavelli*, would necessarily imply vectorial transmission because *M*. *americana* does share habitats with or feed on snakes.

*Trypanosoma minasense* is a trypanosomatid that infects various monkey families [86–90]. According to optical microscopy, it is morphologically similar to *T*. *rangeli* [90] and was considered to be a variant of this species [8]. *Trypanosoma minasense* is distributed from Central America to Argentina [87–88, 91–95], and little is known about its transmission in nature [3, 95–96]. Our results show that the *T*. *minasense* isolated from the *C*. *geoffroyi* monkey clustered with *T*. *bennetti* in the *Megatrypanum* clade in a branch close to *T*. *theileri* [97], reinforcing that at least this *T*. *minasense* sample is not related to *T*. *rangeli*. One possible explanation is that *T*. *minasense* is a diverse taxon or includes more than one species. In fact, there are many remaining open questions concerning *Trypanosoma* species in wild animals.

In conclusion, we observed a unique enzootic scenario in an area with aCD occurrence in the municipality of Guarapari. Here, we also observed that aCD cases can occur even without an enzootic cycle occurring near residential areas. The high trypanosome diversity that exists in such a small, fragmented region of the Atlantic rainforest may be due to the high capacity of bats and *T*. *vitticeps* to act as bioaccumulators of trypanosomes. Even two years after an aCD case occurred, the enzootic scenario did not change. Moreover, *T*. *vitticeps* maintained its vectorial capacity in terms of *T*. *c*. *marinkellei* and *T*. *dionisii*, in addition to four *T*. *c*. *cruzi* DTUs. Understanding this unique scenario will require multidisciplinary foci that include abiotic factors. Ultimately, our study reinforces the plasticity/complexity of the *Trypanosoma* species transmission cycle in nature.

## Supporting information

S1 Table SSU rRNA and gGAPDH GenBank reference sequences used in the phylogenetic analyses of *Trypanosoma* spp. (DOCX)
