# Supplementary material for: High Trypanosoma spp. diversity is maintained by bats and triatomines in Espírito Santo state, Brazil
Source: PLoS One. 2017 Nov 27;12(11):e0188412. doi: 10.1371/journal.pone.0188412 (PMC5703495; doi:10.1371/journal.pone.0188412)
Supplement: S1 Table — (DOCX) [file pone.0188412.s001.docx]

**S1 Table: SSU rRNA and gGAPDH GenBank reference sequences used in phylogenetic analyses of *Trypanosoma* spp.**

| Isolate | Host Origin | GenBank Accession No. | |
| --- | --- | --- | --- |
|  |  | SSU rRNA | gGAPDH |
| *T. c. cruzi* Dm28c | *Didelphis marsupialis* | AF245382 | - |
| *T. c. cruzi* G | Didelphis marsupialis | - | GQ140351 |
| *T. c. cruzi* Y | *Homo sapiens* | - | AB362560 |
| *T. c. cruzi* Sylvio X10 | *Homo sapiens* | AJ009147 | - |
| *T. c. cruzi* Esmeraldocl3 clone2 | *Homo sapiens* | AY785564 | - |
| *T. c. cruzi* MT3663 | *Panstrongylus geniculatus* | AF288660 | JN040971 |
| *T. c. cruzi* MT3869 | *Homo sapiens* | AF303660 | - |
| *T. c. cruzi* TC02 | *Canis familiaris* | JQ912643 | - |
| *T. c. cruzi* CanIII | *Homo sapiens* | - | KT305818 |
| *T. c. cruzi* MT4167 | *Rhodnius brethesi* | AF288661 | - |
| *T. c. cruzi* SO3cl5 clone1 | *Triatoma infestans* | AY785579 | - |
| *T. c. cruzi* SO3cl5 clone 2 | *Triatoma infestans* | AY785580 | - |
| *T. c. cruzi* Nrcl3 | *Homo sapiens* | - | GQ140357 |
| *T. cruzi* CLBR | *Triatoma infestans* | AF245383 | - |
| *T. c. marinkellei* B3 | Phyllostomus discolor | FJ649484 | FJ649495 |
| *T. c. marinkellei* B7 | Phyllostomus discolor | AJ009150 | - |
| *T. c. marinkellei* TryCC 344 | *Carollia perspicillata* | - | GQ140360 |
| *T. c. marinkellei* TryCC 501 | *Carollia perspicillata* | - | GQ140361 |
| *T. erneyi* TCC1294 | *Tadarida* sp. | JN040957 | - |
| *T. erneyi* TCC1946 | *Mops condylurus* | JN040961 | JN040969 |
| *T. erneyi* TCC1934 | *Mops condylurus* | JN040991 | JN040967 |
| *T. erneyi* TCC1936 | *Mops condylurus* | JN040992 | JN040968 |
| *T. dionisii* TCC/USP495 | Carollia perspicillata | FJ001667 | GQ140363 |
| *T. dionisii* P3 | *Pipistrellus pipistrellus* | AJ009151 | FJ649494 |
| *T. dionisii* TryCC211 | *Eptesicus brasiliensis* | - | GQ140362 |
| *T. rangeli* PG | *Homo sapiens* | AJ012417 | KT368805 |
| *T. rangeli* AM80 | *Homo sapiens* | AY491766 | JN040973 |
| *T. rangeli* San Augustin | *Homo sapiens* | - | KT368806 |
| *T. rangeli* 4176 | *Rhodnius brethesi* | EF071580 | - |
| *T. rangeli* Choachi | Rhodnius prolixus | AJ012414 | - |
| *T. rangeli* SC58 | Echimys dasythrix | AY491745 | KT368804 |
| *T. rangeli* TryCC643 | Platyrrinus lineatus | EU867803 | GQ140364 |
| *T. rangeli* RGB (Basel) | Canis familiaris | AJ009160 | - |
| *Trypanosoma* sp. bat | *Rousettus aegyptiacus* | AJ012418 | - |
| *T. vespertilioni* P14 | *Pipistrellus pipistrellus* | AJ009166 | AJ620283 |
| *Trypanosoma* sp. | *Cercopithecus nictitans* | FM202493 | FM164794 |
| *T. conorhini* USP | *Rattus rattus* | AJ012411 | AJ620267 |
| *Trypanosoma* sp. | *Nandinia binotata* | FM202492 | FM164793 |
| *T. minasense* LSTM | *Saimiri boliviensis* | AJ012413 | - |
| *T. leeuwenhoeki* CH 250 | *Choloepus hoffmanni* | AJ012412 | - |
| *Trypanosoma* sp. BACO44 | Artibeus lituratus | KT368797 | KT368800 |
| *Trypanosoma* sp BACO46 | Artibeus lituratus | KT368798 | KT368801 |
| *T. wauwau* CBT68 | *Pteronotus parnellii* | KR653210 | KR653217 |
| *T. wauwau* BMC1069 | *Pteronotus parnellii* | KR653211 | KR653218 |
| *T. wauwau* TCC1022 | Pteronotus sp. | KT368812 | - |
| *T. wauwau* VCT6238 | Pteronotus gymnonotus | KT030840 | - |
| *Trypanosoma* sp. RNMO56 | Trachops cirrhosis | KT368795 | - |
| *Trypanosoma* sp. RNMO63 | Trachops cirrhosus | KT368796 | - |
| *Trypanosoma* sp. 64 | Trichosurus vulpecula | JN315383 | - |
| *Trypanosoma* sp. 17 | Trichosurus vulpecula | JN315382 | - |
| *Trypanosoma* sp. 15 | Trichosurus vulpecula | JN315381 | - |
| *Trypanosoma* sp. G8 | Bettongia penicillata | KC753537 | - |
| *Trypanosoma* sp. H25 | Macropus giganteus | AJ009168 | - |
| *T. livingstonei* 1298 | Rhinolophus landeri | KF192982 | - |
| *T. livingstonei* 1304 | Rhinolophus landeri | KF192983 | - |
| *T. cascavelli* 693 | *Crotalus durissus* | EU095845 | - |
| *T. cascavelli* 632 | *Crotalus durissus* | EU095844 | - |
| *T. gennarii* | *Monodelphis domestica* | KT343360 | - |
| *T. freitasi* | *Didelphis brevicaudata* | MF401951 |  |
| *Trypanosoma* sp. 1052 | *Pseudoboa nigra* | EU095839 | - |
| *Trypanosoma* sp. 910 | Viannamyia tuberculata | EU095838 | - |
| *Trypanosoma* sp. Gecko | Tarentola annularis | AJ620548 | - |
| *T. varani* | *Varanus exanthematicus* | AJ223572 | - |
| *T. cf. varani* | *Python reginus* | AB447493 | AB362559 |
| *T. varani* V54 | *Varanus exanthematicus* | AJ005279 | - |
| *T. scelopori* | *Sceloporus occidentalis* | U67182 | - |
| *T. evansi* E 110 | *Hydrochoerus hydrochaeris* | AJ009154 | - |
| *T. b. gambiense* Tsuaa (clone G) | *Homo sapiens* | AJ009141 | - |
| *Trypanosoma* sp. D30 | *Dama dama* | AJ009165 | - |
| *T. theileri* K127 | *Bos taurus* | AJ009164 | - |
| *T. cyclops* | *Macaca nemestrina* | AJ131958 | - |
| *T. grayi* Crocamp1 | Crocodylus niloticus | KF546526 | - |
| *T. grayi* ANR4 | *Glossina palpalis gambiensis* | AJ005278 | - |
| *T. grayi* | *Glossina gambiensis* | AJ223565 | - |
| *T. minasense* | *Saguinus midas* | AB362412 | - |
| *T. bennetti* | *Falco sparverius* | AJ223562 | - |
| *Trypanosoma* sp. N335 | *Padda aryzivora* | AJ223570 | - |
| *T. corvi* | *Corvus frugilegus frugilegus* | AY461665 | - |
| *T. terrestris* CBT61 | *Tapirus*terrestris | KF586848 | - |
| *Leishmania* sp. MHOM/MQ/92/MAR1 | n/a | AF303938 | - |
| *L. tarentolae* | n/a | M84225 | - |
| *C. fasciculata* | n/a | Y00055 | - |
| *H. samuelpessoai* | n/a | U01016 | - |
| *H. muscarum* | n/a | L18872 | - |
| *H. megaseliae* | n/a | U01014 | - |
| *Phytomonas* sp. TCC297e | n/a | KX219757 | - |
